# Evaluation of a Hydrogel-Based Diagnostic Approach for the Point-of-Care Based Detection of *Neisseria gonorrhoeae*

**DOI:** 10.3390/antibiotics7030070

**Published:** 2018-08-04

**Authors:** Sumudu R Perera, Ali Taheri, Nurul H Khan, Rajinder P Parti, Stephanie Stefura, Pauline Skiba, Jason P Acker, Irene Martin, Anthony Kusalik, Jo-Anne R Dillon

**Affiliations:** 1 Department of Biochemistry Microbiology and Immunology, 2D01 Health Science Building, 107 Wiggins Road, University of Saskatchewan, Saskatoon, SK S7N5E5, Canada; sumudu.perera@usask.ca (S.R.P.); 2 Vaccine and Infectious Disease Organization—International Vaccine Centre, 120 Veterinary Road, Saskatoon, SK S7N5E3, Canada; atahery@gmail.com (A.T.); khanmnh9@gmail.com (N.H.K.); rajparti@gmail.com (R.P.P.); 3 Aquila Diagnostic Systems Inc., 4-034 National Institute for Nanotechnology, 11421 Saskatchewan Drive, Edmonton, AB T6G2M9, Canada; sdc3@ualberta.ca (S.S.); pskiba@ualberta.ca (P.S.); jason.acker@aquiladiagnostics.com (J.P.A); 4 National Microbiology Laboratory, Streptococcus and STI Unit, Public Health Agency of Canada, 1015 Arlington Street, Winnipeg, MB R3E 3R2, Canada; irene.martin@canada.ca; 5 Department of Computer Science, 176 Thorvaldson Building, 110 Science Place, University of Saskatchewan, Saskatoon, SK S7N5C9, Canada; kusalik@cs.usask.ca

**Keywords:** *Neisseria gonorrhoeae*, molecular diagnosis, RT-PCR, hydrogel

## Abstract

Eleven primer pairs were developed for the identification of *Neisseria gonorrhoeae*. The sensitivity and specificity of these primers were evaluated by Real Time (RT)-PCR melt curve analyses with DNA from 145 *N. gonorrhoeae* isolates and 40 other *Neisseria* or non-*Neisseria* species. Three primer pairs were further evaluated in a hydrogel-based RT-PCR detection platform, using DNA extracted from 50 *N. gonorrhoeae* cultures. We observed 100% sensitivity and specificity in the hydrogel assay, confirming its potential as a point-of-care test (POCT) for *N. gonorrhoeae* diagnosis.

## 1. Introduction

*Neisseria gonorrhoeae*, the second most prevalent bacterial sexually transmitted infection (STI) globally, causes 78 million new gonorrhea infections annually [1]. Rates of antimicrobial resistant (AMR) gonorrhea are rising worldwide [2]. Left untreated, gonococcal infections can cause severe and potentially life-threatening disease and reproductive complications, especially in women, including infertility or involuntary death of the fetus [2,3]. With no immunization available, the only way to eliminate infection is with effective antibiotic therapy [4,5]. 

Early diagnosis of infection, preferably at the patient’s first contact with the health care system, is vital for proper control of *N. gonorrhoeae* [6]. Traditional diagnosis of gonorrhea largely relied on microscopy and or culture of the organism from urogenital specimens. Highly sensitive and specific nucleic acid amplification tests (NAATs) have replaced these tests in resource rich settings [7,8,9,10]. In many resource poor settings, either the appropriate laboratory facilities are not available at the primary health care level, or the costs associated with diagnosis are unaffordable. In these instances, patients are treated empirically based on symptoms (i.e., syndromic management) [11,12]. Syndromic management often fails to identify asymptomatic infections and may leave a large population of people at risk for ongoing transmission and severe complications [13,14]. Therefore, it is of the utmost importance to design rapid, easy to perform tests that can provide early diagnosis of *N. gonorrhoeae* even in asymptomatic patients.

An important factor contributing to the rising prevalence of gonorrhea is treatment delay, whereby a patient may wait for a positive diagnostic test result before treatment is initiated [15]. Sometimes, despite the availability of laboratory facilities, the delays in reporting results severely hinders timely treatment [6]. It has been shown by mathematical modeling that a rapid test with a sensitivity as low as 63% can drastically increase the number of patients treated, rather than waiting for a highly sensitive, yet slower test [16]. Likewise, although culture is considered the gold standard for a positive diagnosis, the number of infected individuals diagnosed by a rapid test and in turn successfully treated can outnumber the number of patients who return for treatment following extensive diagnostic procedures such as culturing and more sensitive NAATs [17]. Furthermore, viable organisms are required for culturing. Improper handling and transportation can reduce the viability of *N. gonorrhoeae* leading to false negative results. However, with NAATs, the viability of the organism is not a concern [18,19].

The development of point-of-care tests (POCTs) for an STI such as gonorrhea is recognized as a vital approach to improve timely diagnosis [13,14,20,21,22]. Although POCTs are commercially available for the diagnosis of *N. gonorrhoeae*, many of these tests do not fit the ASSURED criteria important for STI diagnosis tests, such as affordability, sensitivity, specificity, user friendliness, robustness, and rapidness [13,21]. A reliable and rapid diagnostic test for *N. gonorrhoeae* would be a critical advancement in diagnostic capability. 

The portable hydrogel-based RT-PCR method, developed by Aquila Diagnostic Systems Inc. Edmonton AB, Canada, is a rapid diagnostic platform utilizing Real Time Polymerase Chain Reaction (RT-PCR) melt curve analysis to amplify nucleic acids directly in clinical specimens and report the presence of specific genetic targets [23]. This system has successfully detected blood-borne infectious diseases such as malaria, BK virus, and herpes simplex virus [23,24,25]. We developed and evaluated eleven diagnostic primer pairs for the identification of *N. gonorrhoeae.* We then evaluated three of these primers with the hydrogel-based detection method to assess the potential of this method for further development as a POCT.

## 2. Results

### 2.1. Evaluation of N. gonorrhoeae Diagnostic Primer Pairs

From the eleven primer pairs tested (Table 1), nine primer pairs (3, 8-3, 8-4, 13, 16, 17-1, 17-2, 21-5, 31-2) amplified every *N. gonorrhoeae* isolate tested (*n* = 130) (Table 2). Primer pair 2 did not amplify one of the 130 *N. gonorrhoeae* isolates. Primer pair 31-3 did not amplify 28 of the 130 *N. gonorrhoeae* isolates. Primer pairs 2, 3, 8-4, 16, 17-1, 17-2, 21-5, 31-2, and 31-3 did not amplify any of the non-*N. gonorrhoeae* or non-*Neisseria* species. However, primer pair 8-3 amplified DNA from *Neisseria flava*, *Neisseria lactamica*, and *Neisseria polysacchareae*. Primer pair 13 amplified DNA from *N. flava*, *Neisseria subflava*, *Neisseria mucosa*, *N. lactamica*, *Neisseria perflava*, *Neisseria flavescens*, and *N. polysacchareae* (Table 2). The limit of detection of the primers was assessed by serially diluting (10 ng/µL to 0.000001 ng/µL) genomic DNA from *N. gonorrhoeae* strain WHO-M. After 30 cycles of PCR, all the primers were able to detect gonococcal concentrations as low as 0.001 ng/uL. 

### 2.2. Optimization of the Hydrogel System and Comparison with RT-PCR Methods

The ability of primers 3, 17-1, and 21-5 to identify genomic DNA from *N. gonorrhoeae* strains WHO F, WHO P, WHO G, WHO K, and WHO N in the hydrogel system was analyzed using RT-PCR (Table 3). To optimize the performance of the hydrogel system for *N. gonorrhoeae*, different parameters for hydrogel and RT-PCR were assessed. In testing for the most effective concentration of SYBR-Green, we determined that with 1× SYBR-Green (pre-mixed in the desiccated gel), primer pair 3 produced melt curve temperatures (Tm values) similar to previously established Tm values (80.5 °C) with control methods. However, with primer pairs 17-1 and 21-5, 2× SYBR-Green was required for established Tm values (85.0 °C). Therefore, 2× SYBR-Green was chosen for subsequent experiments. Further, with our primer pairs, we determined that optimal amplification occurred with 30 PCR cycles, which differs from the hydrogel manufacturer’s recommendation of 40 cycles.

The performance of the hydrogel method with increasing concentrations of DNA (50, 70, or 100 ng/µL) was assessed using genomic DNA from 50 *N*. *gonorrhoeae* positive cultures (Table 4: Panel 2), WHO *N. gonorrhoeae* reference strains (*n* = 5; positive control) and non-*N. gonorrhoeae* strains (*n* = 4; negative control). Based on the melt curve (Tm) temperatures (primer pair 3, 80.5 °C; primer pairs 17-1 and 21-5, 85.0 °C), primer pair 3 with 50 ng/µL DNA identified 44 *N. gonorrhoeae* isolates. With 70 ng/µL DNA, primer pair 3 identified 46 isolates and with 100 ng/µL DNA identified all 50 *N. gonorrhoeae* isolates (Table 5). Thus, primer pair 3 had 88%, 92% and 100% sensitivity with 50, 70 and 100 ng/µL DNA respectively. The specificity of primer pair 3 was 100% with all DNA concentrations tested. Primer pair 17-1, irrespective of the DNA concentration tested, identified all 50 *N. gonorrhoeae* isolates and had 100% sensitivity and specificity. Primer pair 21-5 identified 47 *N. gonorrhoeae* isolates with 50 ng/µL DNA and had a sensitivity of 94% and a specificity of 100%. With 70 ng/µL and 100 ng/µL DNA, primer pair 21-5 identify all 50 isolates and had 100% sensitivity and specificity. Primer pairs 3 and 17-1 in the control RT-PCR method identified all 50 *N. gonorrhoeae* isolates and had a sensitivity of 100%. Primer pair 21-5 identified 48 isolates with a sensitivity of 96%. One non-*Neisseria* species (*Escherichia coli*) was incorrectly identified as *N. gonorrhoeae* positive by primer pair 3 in the RT-PCR control method, thereby giving a specificity of 67% (Table 5). With primer pairs 17-1 and 21-5, all the negative control isolates (*E. coli*, *Lactobacillus jensenii*, *Neisseria elongata*, *Neisseria subflava*, and *Salmonella enterica* serovar Typhimurium) were correctly ascertained as *N. gonorrhoeae* negative, giving a specificity of 100%.

With the hydrogel method and primer pair 3, 33 samples had positive threshold cycle (Ct) values when using 70 ng/µL DNA. However, with 50 ng/µL and 100 ng/µL DNA, none of the samples had positive Ct values. Similarly, with primer pairs 17-1 and 21-5, all 50 samples had negative Ct values. In contrast, when using the control RT-PCR method and primer pair 3, all 50 samples had positive Ct values. Likewise, with primer pair 17-1, all 50 samples had positive Ct values. With primer pair 21-5, 49 samples had positive Ct values. To consider if the hydrogel system was interfering with the fluorescence reading, extra, fresh SYBR-Green (commercially made 2× SYBR-Green) was added to the reaction mixture of samples with the hydrogel method. These reaction mixtures pre-contained 2× SYBR-Green incorporated in to the desiccated gel. The addition of total 4x and fresh SYBR-Green significantly improved the results from the hydrogel method and produced Ct values similar to that obtained with RT-PCR control method (Table 5).

## 3. Discussion

We developed and evaluated novel primers in a hydrogel platform as a potentially useful POCT for *N. gonorrhoeae* diagnosis. This system is rapid and requires minimal sample preparation. The hydrogel system introduces a state-of-the-art portable diagnostic approach to bringing a detection platform to clinicians and to remote locations, as this system contains desiccated primers, polymerases and other PCR components, thereby enabling the prompt delivery of results. Only the patient sample would need to be added to the system prior to PCR amplification. As such, this system does not require a cold chain (i.e., refrigeration) to preserve the PCR reagents, a major obstacle to delivering diagnostic tests to remote locations. 

A recent review evaluated POCTs for *N. gonorrhoeae* [13]. The tests evaluated include lateral flow immunochromatographic test (ICT) formats, i.e., the Binax NOW Gonorrhoea Test (Inverness), the GC Check (PATH, Seattle, Washington, USA) and, the ACON format tests (ACON Laboratories, San Diego, California, USA); an optical immunoassay test (OIA), the BioStar (ThermoFisher/BioStar, Boulder, Colorado, USA); and a nucleic acid amplification test, the GeneXpert CT/NG Assay (Cepheid, Sunnyvale, California, USA). The performance of the ICTs was variable with sensitivities ranging from 94%, 70%, and 12.5% and specificities from 96%, 97%, and 99.8% for Binax NOW, GC Check and ACON format tests, respectively. The OIA BioStar test had a sensitivity of 60% and a specificity of 89%. The ICTs and the OIA had 5 or more steps and took 25 to 40 minutes to obtain results [13,14]. These tests generally fail to meet the high levels of sensitivity and specificity required for a POCT. The GeneXpert CT/NG Assay is the only commercial NAAT-based POCT for *N. gonorrhoeae* diagnosis and has high sensitivity (95.6–98%) and specificity (99.9–100%) which is dependent on the specimen type [13,30,31,32,33]. This assay requires 3 steps and takes 90 minutes to produce results [31]. The limiting factor about considering the GeneXpert as a POCT is affordability and portability of the equipment. 

The currently available United States Food and Drug Administration approved commercial NAAT systems for *N. gonorrhoeae* identification primarily rely on a single target detection strategy [34]. These tests include the Abbott Real Time CT/NG assay which targets a 48bp sequence within the *N. gonorrhoeae opa* gene (Abbott Laboratories, Abbott Park, IL, USA), the Aptima COMBO 2 assay and the Aptima GC assay which target specific regions within *N. gonorrhoeae* 16S rRNA (Hologic/Gene-Probe Inc., San Diego, CA, USA), the BD ProbeTec ET CT/GC Amplified DNA assay and BD ProbeTec Qx GC Amplified DNA Assay which target the chromosomal pilin gene-inverting protein homologue (Becton Dickinson and Company, Sparks, MD, USA), the Cobas 4800 CT/NG which targets the DR-9 region (Roche Diagnostics, Indianapolis, IN, USA) and the GeneXpert CT/NG Assay (Cepheid, Sunnyvale, CA, USA) which uses two non-contiguous chromosomal targets (NG2 and NG4) of *N.* gonorrhoeae [7,30,31,34,35]. 

The hydrogel method we evaluated had 100% sensitivity and specificity using DNA from *N. gonorrhoeae* cultures. The system remains to be tested using clinical specimens of *N. gonorrhoeae* such as urines or self-collected vaginal or rectal swabs. One complication that arose with the hydrogel method was that, despite having a positive *N. gonorrhoeae* diagnosis by melt curve (Tm) analysis, most samples contained negative threshold cycle (Ct) values. Therefore, we considered that the hydrogel system was interfering with the fluorescence reading, thereby giving Ct values lower than the threshold value (i.e., negative Ct values). Addition of extra, fresh 2× SYBR-Green to the hydrogel reaction mixture pre-containing 2× SYBR-Green, for a final concentration of 4× SYBR-Green resulted in favorable Ct values. The hydrogel has been successfully used for the detection of other pathogens such as Herpes simplex virus from raw genital swabs, *Plasmodium falciparum*, BK virus, human platelet antigen 1 (HPA_1_), and fibroblast growth factor receptor 2 (FGFR_2_) from unprocessed blood samples [23,24,25]. Furthermore, the <2-hour time frame from specimen collection to results makes this test a potentially much simpler and user-friendly second molecular POCT.

The genomic targets of three of our primer pairs have partial homology with genomic targets used in two commercial assays. Primer pair 2 has partial homology with the genomic region used in the cobas 4800 CT/NG Test (i.e., DR-9 region) and primer pairs 8-3 and 13 have partial homology with the genomic targets used in the BD ProbeTec Amplified DNA Assays (i.e., chromosomal pilin gene-inverting protein homologue). In our assay, primer pair 2 did not amplify one *N. gonorrhoeae* isolate. Furthermore, cross-reactivity of primer pairs 8-3 and 13 with 3 and 7 other non-gonococcal *Neisseria* species respectively was observed. Cross-reactivity with *Neisseria subflava* and *Neisseria lactamica* was also reported for the BD ProbeTec GC Q^x^ Amplified DNA Assay [8]. 

## 4. Materials and Methods 

### 4.1. Bacterial Strains

To determine the sensitivity and specificity of primer pairs developed by us for the identification of *N. gonorrhoeae*, DNA from 145 *N. gonorrhoeae* isolates was obtained from the Dillon Culture Collection and represented isolates from diverse geographical areas [26,27,28] (Table 4: Panel 1). These isolates included the World Health Organization (WHO) *N. gonorrhoeae* reference isolates M, L, F, B, O, C, P, K, G, and N [29]. A random collection of 40 other *Neisseria* and non-*Neisseria* species were obtained from the National Microbiology Laboratory (NML), Winnipeg MB, Canada (Table 4: Panel 1). 

For experiments evaluating the hydrogel platform, DNA from 50 cultured *N. gonorrhoeae* isolates was used (Table 4: Panel 2 is a sub-panel of Panel 1). WHO *N. gonorrhoeae* reference strains WHO-F, P, G, K and N were used as positive controls. Five non-*N. gonorrhoeae* and non-*Neisseria* species (*E. coli*, *L. jensenii*, *N. elongata*, *N. subflava*, and *S. enterica* serovar Typhimurium) were used as negative controls. 

### 4.2. DNA Extraction

DNA extraction was performed with a QIAamp DNA Mini Kit (#51306 Qiagen Canada) according to manufacture’s instructions. DNA was quantified using a NanoDrop 1000 Spectrophotometer (Thermo Fisher Scientific Inc., Wilmington DE, USA) and the final concentration was adjusted to 50, 70, or 100 ng/µL for evaluating the hydrogel method.

### 4.3. Design of Diagnostic Primer Pairs

Non-homologous sequence regions between *N. gonorrhoeae* FA1090 and *N. meningitidis* (MC58, FAM18 and Z2491) were extracted from the *N. gonorrhoeae* FA1090 genome. These sequences were then BLASTed against 25 in-house sequenced *N. gonorrhoeae* genomes and 15 *N. gonorrhoeae* genomes available at the Broad Institute database. Sequences which were positive in this BLAST search (50 sequences) were then further aligned against the NCBI non-redundant (nr) database using the BLAST program. Regions which were only found in *N. gonorrhoeae* were filtered for further analysis. Targets for all primer pairs are present in multiple copies in the *N. gonorrhoeae* FA1090 genome and in turn increase the sensitivity of detection even at lower DNA concentrations. Based on these findings, eleven primer pairs were designed using Primer-BLAST [36] (Table 1). Potential cross-reactivity of the primer pairs was evaluated *in silico* using primer-BLAST software against the NCBI nr database. Primer pairs were tested *in vitro* for their sensitivity and specificity with *N. gonorrhoeae*, non-*N. gonorrhoeae* and non-*Neisseria* species.

### 4.4. Limit of Detection of Primers

RT-PCR reactions contained 5 µL of 2× SYBR-Green master mix (Cat # 4472912, Life Technologies Inc.), 0.25 µL of each primer (10 µM), 1 µL of DNA (50 ng/µL), and 3.5 µL PCR-grade water in a total 10 µL reaction volume. Initial holding and activation of DNA polymerase was at 50 °C for 2 minutes. RT-PCR was performed for 40 cycles of 95 °C for 20 seconds and 60 °C for 40 seconds. A post-PCR melt curve was performed between 60 °C to 95 °C with 0.3 °C temperature increments. Limit-of-detection assays, using serial dilutions (100 ng/µL to 0.00001 ng/µL) of DNA from *N. gonorrhoeae* reference strains WHO F, P, G, K, and N, were used to ascertain the sensitivity of each primer pair. DNA from 40 non-*N. gonorrhoeae* and non-*Neisseria* species was also used to test the specificity of each primer pair. 

### 4.5. Real Time PCR and Hydrogel Methods 

To compare hydrogel and RT-PCR methods, DNA was amplified using three *N. gonorrhoeae* diagnostic primer pairs (3, 17-1, and 21-5) and a SYBR Select master mix. For the RT-PCR control method, each reaction contained 2 µL DNA (50, 70, or 100 ng/µL), 1.5 µL of each primer (10 µM) and 5 µL 2× SYBR-Green master mix in a total 10 µL reaction volume. For the hydrogel method, each reaction contained 1 µL DNA (50, 70, or 100 ng/µL), 0.38 µL of each primer (10 µM), and 8.24 µL PCR-grade water. 2× SYBR-Green and primers were pre-mixed with the hydrogel. DNA and water were added to the hydrogel immediately before the start of the RT-PCR assay. Initial holding and activation of DNA polymerase was at 50 °C for 2 minutes. RT-PCR was then performed for 30 cycles of 95 °C for 20 seconds and 60 °C for 40 seconds. A post PCR melt curve was performed between 60 °C to 95 °C with 0.3 °C temperature increments. 

Various RT-PCR conditions were tested to optimize the hydrogel method. The final assay conditions included: 30 PCR cycles, 60 °C annealing temperature, 60–95 °C melt curve temperature range, 0.3 °C image taking temperature increments, and 2× SYBR-Green concentration. 

## 5. Conclusions

The hydrogel method coupled with the primers discussed in this work can meet the ideal rapid test (ASSURED) criteria established by WHO for STI diagnosis tests; Affordable, Sensitive, Specific, User-friendly, Robust and Rapid, Equipment-free, and Deliverable to end users [21]. Although the hydrogel system was evaluated using pure cultures of *N. gonorrhoeae*, with further optimization the hydrogel diagnostic method with these primers can be used as a POCT for the diagnosis of gonococci from clinical specimens enabling rapid, reliable, and efficient diagnosis of *N. gonorrhoeae*. Furthermore, multiplex primer pairs can be integrated into the hydrogel platform enabling simultaneous diagnosis of *N. gonorrhoeae* and characterization of its AMR profile to different antimicrobials.

## Figures and Tables

**Table 1:** Primer sequences used in this study for detection of *N. gonorrhoeae*, their respective targets and copy numbers present in the *N. gonorrhoeae* genome.

Primer ID (product length)	Sequence (5’->3’) ^a^	Target Gene ^b^	No. Targets in FA1090 ^c^	Similar Targets from Commercial NAATs
Primer 2 (208bp)		NGO1620, NGO0469, NGO1126	3	Cobas 4800 CT/NG
Forward	TCTGCTTTCTTGGTGGGCGA			
Reverse	AGGCGATCCGGAAATGCTGA			
Primer 3 (139bp)		NGO05940, NGO06090, NGO06650, NGO1642	4	-
Forward	TATGGGGGTTCCTTCGCACC			
Reverse	CAGACGGTTGCGGGTTCTTG			
Primer 8-3 (132bp)		NGO0773, NGO1200, NGO1703, NGO1137, NGO1164, NGO1262, NGO1641	7	BD ProbeTec GC Qx
Forward	CAGAAGCCTACGGACGAGCA			
Reverse	CGCATATGCTTTGGCCGCTT			
Primer 8-4 (180bp)		NGO0773, NGO1200, NGO1703, NGO1137, NGO1164, NGO1262, NGO1641	7	-
Forward	TCACGGATGACCGCAGCATA			
Reverse	AGACGCTTCACGCCTTCCTT			
Primer13 (138bp)		NGO0773, NGO1137, NGO1703, NGO1164, NGO1200, NGO1262, NGO1641	7	BD ProbeTec GC Qx
Forward	GCGTAACGCCGTAGGATTGGA			
Reverse	CCCAAGCTTTTCAACCGGTCC			
Primer16 (93bp)		NGO1131, NGO1209	2	-
Forward	CGGAACAAGCGTTTTTCAGCG			
Reverse	TCTTTGGCTTGTCCGGGTGT			
Primer 17-1 (73bp)		NGO1638, NGO0487, NGO1108	3	-
Forward	TCCGAAACACGCAAACCGAAA			
Reverse	TAGCCCGGGTTGGTATTGCC			
Primer 17-2 (82bp)		NGO1638, NGO0487, NGO1108	3	-
Forward	ACACGCAAACCGAAACCGTC			
Reverse	GCGCGGTTTTTGTAATAGCCC			
Primer 21-5 (101bp)		NGO1085, NGO1652	2	-
Forward	GCACGAAACCCGTCCAATCC			
Reverse	CAAGACATGCGGCTATGCGG			
Primer 31-2 (188bp)		NGO0480, NGO1113, NGO1631	3	-
Forward	AAAATCGCGCCGGGTTTGAA			
Reverse	AGCTTATCCGCAGCGGTTCT			
Primer 31-3 (275bp)		NGO0480, NGO1113, NGO1631	3	-
Forward	AAAAAGCCCGTCGGGTCAGA			
Reverse	AACCCGAAGAATCGGAGCCA			

^a^ The primer sequences presented in this manuscript are the subject of a United States utility patent (#62/088,332). ^b^ Locus Tag ID in the NCBI database. ^c^ Number of targets on *N. gonorrhoeae* FA1090 genome.

**Table 2:** Evaluation of eleven primer pairs using *N. gonorrhoeae*, non-*N. gonorrhoeae* and non-*Nesseria* species.

Bacterial Species	No. of Isolates	Positive Amplifications										
		2 ^a^	3	8-3 ^b^	8-4	13 ^b^	16	17-1	17-2	21-5	31-2	31-3
*N. gonorrhoeae*	130	129	130	130	130	130	130	130	130	130	130	102
*N. flava*	2	0	0	**1**	0	**2**	0	0	0	0	0	0
*N. subfla va*	2	0	0	0	0	**1**	0	0	0	0	0	0
*N. elongata*	2	0	0	0	0	0	0	0	0	0	0	0
*N. mucosa*	3	0	0	0	0	**1**	0	0	0	0	0	0
*N. lactamica*	4	0	0	**2**	0	**2**	0	0	0	0	0	0
*N. perflava/sicca*	5	0	0	0	0	**3**	0	0	0	0	0	0
*N. flavescents* ^c^	2	0	0	0	0	**1**	0	0	0	0	0	0
*N. polysacchareae*	4	0	0	**1**	0	**2**	0	0	0	0	0	0
Other *Neisseria* species ^d^	6	0	0	0	0	0	0	0	0	0	0	0
Non-*Neisseria* species ^e^	10	0	0	0	0	0	0	0	0	0	0	0

^a^ Primers amplified regions with partial homology to DR9 repeats used in cobas 4800 CT/NG Test (Roche Molecular Diagnostics, Pleasanton CA, USA). ^b^ Primers amplified regions with partial homology to targets used in BD ProbeTec GC Qx Amplified DNA Assay (Becton Dickinson and Company, Franklin Lakes NJ, USA). ^c^ *Neisseria flavescens*. ^d^ *Neisseria animaloris* (1), *Neisseria cinerea* (2), *Neisseria menengitidis* (1), *Neisseria wadsworthii* (1), *Neisseriaweaverii* (1). ^e^ *Enterococcus faecalis* (1), *Enterococcus faecium* (1), *Escherichia coli* (1), *lebsiella oxytoca* (1), *Lactobacillus jensenii* (1), *Moraxella catarrhalis* (1), *Pseudomonas aeruginosa* (1), *Salmonella enterica* serovar Typhimurium (1), *Staphylococcus aureus* (1), and *Staphylococcus epidermis* (1).

**Table 3:** The effect of SYBR-Green concentration on melt curve temperature (Tm values) of five *N. gonorrhoeae* strains, with primer pairs 3, 17-1, and 21-5.

Strain	Melt Curve Temperature (°C)					
	Primer Pair 3 ^a^		Primer Pair 17-1 ^a^		Primer Pair 21-5 ^a^	
	1× SYBR-Green	2× SYBR-Green	1× SYBR-Green	2× SYBR-Green	1× SYBR-Green	2× SYBR-Green
WHO-F	80.49	81.52	83.90	84.64	84.05	84.84
WHO-G	80.78	81.82	84.05	84.64	83.90	84.79
WHO-K	80.49	81.97	84.05	84.49	84.20	84.79
WHO-N	80.63	81.82	83.90	84.49	84.05	84.79
WHO-P	80.93	81.97	83.90	84.64	84.05	84.79

^a^ Expected Tm values for primer pair 3—80.5 °C; primer pair 17-1—85.0 °C; primer pair 21-5—85.0 °C.

**Table 4:** *N. gonorrhoeae*, non-*N. gonorrhoeae*, and non-*Neisseria* isolates used in this study.

Isolate Selection	Organisms	Geographic Source	No	References
Panel 1	*N. gonorrhoeae*	Saskatchewan	86	[26,27]
		USA	13	Dillon Culture Collection
		China	8	[28]
		WHO	10	[29]
		South America and the Caribbean	28	Dillon Culture Collection
	Non-*N. gonorrhoeae* ^b^	Canada	30	NML ^a^
	Non-*Neisseria* species ^b^	Canada	10	NML
Panel 2	*N. gonorrhoeae*	Saskatchewan	35	[26,27]
		China	6	[28]
		South America and the Caribbean	9	Dillon Culture Collection
		WHO	5	[29]
	Non-*N. gonorrhoeae* ^c^	Canada	2	NML
	Non-*Neisseria* species ^c^	Canada	3	NML

^a^ National Microbiology Laboratory. ^b^ One isolate of: *Enterococcus faecalis, Enterococcus faecium, Escherichia coli, Klebsiella oxytoca, Lactobacillus jensenii, Moxarella catarrhalis, Neisseria animaloris, Neisseria cinerea, Neisseria elongata, Neisseria flava, Neisseria lactamica, Neisseria menengitidis, Neisseria mucosa, Neisseria perflava, Neisseria polysacchareae, Neisseria sicca, Neisseria subflava, Neisseria wadsworthii, Neisseria weaverii, Pseudomonas aeruginosa, Salmonella enterica serovar Typhimurium, Staphylococcus aureus*, and *Staphylococcus epidermis.* ^c^ One isolate of: *E. coli, L. jensenii, N. elongata, N. subflava,* and *S. enterica* serovar Typhimurium.

**Table 5:** Evaluation of hydrogel and RT-PCR methods using varying concentrations of *N. gonorrhoeae* DNA (*n* = 50) ^a^ and diagnostic primer pairs 3, 17-1, and 21-5.

Primer Pair	Method	DNA Conc. (ng/µL)	No *N. gonorrhoeae* Isolates Identified as per Tm value (*n* = 50) ^b^	Positive Ct values (*n* = 50) ^b^	Sensitivity (%)	Specificity (%)
3	Hydrogel	50	44	0	88	100
3	Hydrogel	70	46	33	92	100
3	Hydrogel	100	50	0	100	100
3	Hydrogel+4× SYBR ^d^	100	50	50	100	100
3	RT-PCR control	70	50	50	100	67 ^c^
17-1	Hydrogel	50	50	0	100	100
17-1	Hydrogel	70	50	0	100	100
17-1	Hydrogel	100	50	0	100	100
17-1	RT-PCR control	70	50	50	100	100
21-5	Hydrogel	50	47	0	94	100
21-5	Hydrogel	70	50	0	100	100
21-5	Hydrogel	100	50	0	100	100
21-5	RT-PCR control	70	48	49	96	100

^a^ Controls—WHO *N. gonorrhoeae* reference strains (*n* = 5; positive control) and non-*N. gonorrhoeae* and non-*Neisseria* strains (*n* = 4; negative control). ^b^ Number of *N. gonorrhoeae* isolates identified as per the melt curve temperature (Tm) and threshold cycle (Ct) values. Isolates with negative Ct values had a positive melt curve temperature (Tm). ^c^ One Non-*N. gonorrhoeae* isolate identified as *N. gonorrhoeae* positive. ^d^ Hydrogel method pre-contained 2× SYBR-Green dye. For this assay, extra, fresh 2× SYBR-Green dye was added for a final concentration of 4× SYBR-Green dye.

## Supplementary Materials

The following are available online at http://www.mdpi.com/2079-6382/7/3/70/s1, Table S1: Primer sequences used in this study for detection of *N. gonorrhoeae*, their respective targets and copy numbers present in the *N. gonorrhoea*e genome.
