# Supplementary material for: Evaluation of a Hydrogel-Based Diagnostic Approach for the Point-of-Care Based Detection of Neisseria gonorrhoeae
Source: Antibiotics (Basel). 2018 Aug 4;7(3):70. doi: 10.3390/antibiotics7030070 (PMC6164196; doi:10.3390/antibiotics7030070)
Supplement: Supplementary file 1 [file antibiotics-07-00070-s001.pdf]

Table S1: Primer sequences used in this study for detection of *N. gonorrhoeae*, their respective targets and copy numbers present in the *N. gonorrhoeae genome*

Primer ID (product length)	Sequence (5'->3')	Target Gene[b]	No Targets in FA1090[c]	Similar Targets from Commercial NAATs
Primer 2 (208bp)<br>Forward<br>Reverse	 TCTGCTTTCTTGGTGGGCGA<br>AGGCGATCCGGAAATGCTGA	NGO1620, NGO0469, NGO1126	3	Cobas 4800 CT/NG
Primer 3 (139bp)<br>Forward<br>Reverse	 TATGGGGGTTCCTTCGCACC<br>CAGACGGTTGCGGGTTCTTG	NGO05940, NGO06090, NGO06650, NGO1642	4	-
Primer 8-3 (132bp)<br>Forward<br>Reverse	 CAGAAGCCTACGGACGAGCA<br>CGCATATGCTTTGGCCGCTT	NGO0773, NGO1200, NGO1703, NGO1137, NGO1164, NGO1262, NGO1641	7	BD ProbeTec GC Qx
Primer 8-4 (180bp)<br>Forward<br>Reverse	 TCACGGATGACCGCAGCATA<br>AGACGCTTCACGCCTTCCTT	NGO0773, NGO1200, NGO1703, NGO1137, NGO1164, NGO1262, NGO1641	7	-
Primer13 (138bp)<br>Forward<br>Reverse	 GCGTAACGCCGTAGGATTGGA<br>CCCAAGCTTTTCAACCGGTCC	NGO0773, NGO1137, NGO1703, NGO1164, NGO1200, NGO1262, NGO1641	7	BD ProbeTec GC Qx
Primer16 (93bp)		NGO1131, NGO1209	2	-

Forward	CGGAACAAGCGTTTTTCAGCG			
Reverse	TCTTTGGCTTGTCCGGGTGT			
Primer 17-1 (73bp)		NGO1638, NGO0487, NGO1108	3	-
Forward	TCCGAAACACGCAAACCGAAA			
Reverse	TAGCCCGGGTTGGTATTGCC			
Primer 17-2 (82bp)		NGO1638, NGO0487, NGO1108	3	-
Forward	ACACGCAAACCGAAACCGTC			
Reverse	GCGCGGTTTTTGTAATAGCCC			
Primer 21-5 (101bp)		NGO1085, NGO1652	2	-
Forward	GCACGAAACCCGTCCAATCC			
Reverse	CAAGACATGCGGCTATGCGG			
Primer 31-2 (188bp)		NGO0480, NGO1113, NGO1631	3	-
Forward	AAAATCGCGCCGGGTTTGAA			
Reverse	AGCTTATCCGCAGCGGTTCT			
Primer 31-3 (275bp)		NGO0480, NGO1113, NGO1631	3	-
Forward	AAAAAGCCCGTCGGGTCAGA			
Reverse	AACCCGAAGAATCGGAGCCA			

[a]The primer sequences presented in this manuscript are the subject of a United States utility patent (#62/088,332)

[b]Locus Tag ID in the NCBI database

[c]Number of targets on *N. gonorrhoeae* FA1090 genome
